# Tumour–Stroma Ratio as a Predictive Biomarker for Neoadjuvant Therapy Efficacy in Rectal Cancer

**DOI:** 10.3390/cancers18132089

**Published:** 2026-06-27

**Authors:** Jonathan P. Callaghan, Caroline R. Cartlidge, Kenal Patel, Nicholas P. West

**Affiliations:** Division of Pathology and Data Analytics, Leeds Institute of Medical Research, University of Leeds, Leeds LS9 7TF, UK; j.p.callaghan@leeds.ac.uk (J.P.C.);

**Keywords:** tumour–stroma ratio, tumour cell density, predictive biomarker, pathological response, neoadjuvant, radiotherapy, immunotherapy, chemotherapy, rectal cancer, colorectal cancer, tumour microenvironment

## Abstract

**Background**: The treatment of rectal cancer frequently involves a multimodal approach, including neoadjuvant therapy prior to surgery in patients with locally advanced disease. However, the response to such treatment is variable. Robust biomarkers to predict neoadjuvant therapy response represent an unmet clinical need; they could help to stratify patients for organ preservation strategies or treatment intensification. The tumour–stroma ratio (TSR) is an established prognostic marker that has recently gained attention for its potential predictive value when assessed in pre-treatment biopsies. **Objective**: This narrative review critically evaluates the existing evidence regarding TSR as a predictive biomarker for neoadjuvant therapy response in rectal cancer. **Results**: Emerging evidence from retrospective studies of large cohorts suggests that stroma-high tumours often demonstrate resistance to standard neoadjuvant chemoradiotherapy, resulting in lower major pathological response rates. Conversely, some smaller studies report no significant association between biopsy TSR and treatment efficacy. This conflicting evidence could be attributable to methodological heterogeneity, including inconsistent definitions, varying measurement techniques (manual versus automated), and mixed patient cohorts. The predictive value of TSR appears to be neoadjuvant regimen-specific, with stroma-high phenotypes interacting differently with treatments like short-course radiotherapy or intensified chemotherapy. **Conclusions**: TSR is a simple, biologically plausible, and readily assessable promising biomarker with apparently predictive as well as prognostic potential. It is likely to represent a regimen-specific predictor rather than a universal marker of resistance to neoadjuvant therapy in rectal cancer. Future clinical translation will require standardised, AI-driven quantification and robust prospective clinical validation.

## Simple Summary

Rectal cancer is often treated with drugs and/or radiation before surgery, but patient responses vary widely. There is a strong need for ways to predict how well a patient might respond to these therapies, allowing for treatment personalisation. In the context of diagnostic biopsies, this review explores the tumour–stroma ratio, which measures the amount of connective tissue (stroma) relative to cancer cells. We aimed to critically evaluate how this measurement is undertaken and the existing evidence for its use in predicting treatment success in rectal cancer. Our findings suggest that tumours with high amounts of stroma may often resist standard treatments but might respond to other specific or intensified therapies. While the tumour–stroma ratio is a simple and promising marker, it may predict responses differently depending on the particular treatment given. In the future, by standardising how it is measured, likely using artificial intelligence, the tumour–stroma ratio in diagnostic biopsies might be used to guide personalised care in patients with rectal cancer.

## 1. Introduction: The Imperative for Predictive Biomarkers in Rectal Cancer

Colorectal cancer is a leading cause of cancer-related mortality worldwide [1]. In rectal cancer, the standard of care often involves a multimodal treatment approach including neoadjuvant chemotherapy, radiotherapy, and/or immunotherapy followed by surgical resection. While this approach has significantly improved local control, patient response to neoadjuvant therapy is highly variable [2,3], ranging from a pathological complete response (pCR), where no viable tumour cells remain in the surgical specimen, to the complete absence of histopathological tumour regression, where patients suffer the potential toxicity of treatment without any clear oncological benefit. Currently, robust biomarkers to predict the response to neoadjuvant therapy prior to treatment and stratify patients into appropriate management strategies are lacking [4,5]. Patients who are predicted to achieve a pCR may be candidates for organ preservation strategies, sparing them from radical surgery. Conversely, identifying non-responders upfront could allow for either immediate surgery or alternative treatments including intensification. The identification of predictive biomarkers for neoadjuvant therapy therefore remains an urgent unmet need in the management of rectal cancer.

While several candidate predictive biomarkers, including tissue-based markers, have been investigated, implementation into routine clinical practice has been limited for reasons such as varied study designs, a lack of reproducibility, or failure to validate [4,5]. In this context, the tumour–stroma ratio (TSR) and related tumour cell density (TCD)—assessed on standard haematoxylin and eosin (H&E)-stained slides—have emerged as promising candidates. The prognostic value of TSR has been demonstrated across various cancer types, with a meta-analysis showing that stroma-high solid tumours are associated with poor overall and disease-free survival [6]. This is also observed in colorectal cancer, where TSR, which quantifies the relative proportion of tumour to stroma within the tumour microenvironment (TME), is firmly established as a prognostic marker in resection specimens [7,8,9]. The large, international UNITED study recently validated this prognostic relevance [8]. Consequently, the Union of International Cancer Control has included TSR as a prognostic factor in the ninth edition of the TNM classification [10], encouraging pathologists to routinely assess it in resection specimens. For patients receiving neoadjuvant treatment, the post-treatment pathological response (measured by the tumour regression grade or residual TCD, for example) is prognostic across radiotherapy [3,11], chemotherapy [2], and chemoradiotherapy regimens [12]. TSR assessed in surgical resection specimens also shows utility as a predictive biomarker in the adjuvant therapy setting [13], but this is of less relevance to neoadjuvant treatment.

Recently, attention has shifted to the predictive value of TSR assessed in pre-treatment diagnostic biopsies. Without neoadjuvant treatment, the TSR assessed on preoperative colorectal cancer biopsies has been shown to strongly predict the TSR in matched surgically resected specimens [14]. Therapy is likely to alter the tumour stroma, but emerging evidence suggests that the stromal composition of the biopsy may serve as a surrogate marker for therapeutic sensitivity, with stroma-high tumours potentially exhibiting resistance to standard chemoradiation. Precedence for the potential predictive utility of biopsy-derived TSR exists in other solid tumours, such as oesophageal cancer [15]. In colorectal cancer, the literature is evolving but is hampered by varying definitions, some null findings, and methodological heterogeneity. This review will focus on biopsy-based assessment and critically evaluate the existing evidence for TSR as a predictive biomarker for neoadjuvant therapy response in rectal cancer. Because neoadjuvant chemoradiotherapy (nCRT) is used more routinely in rectal cancer than colon cancer, the studies in this area predominantly relate to rectal cancer. Therefore, this review focuses primarily on rectal cancer, while drawing on the wider colorectal literature where relevant.

## 2. Methodology and Definitions

### 2.1. Search Strategy

As a narrative review, articles were selected to provide a broad translational overview of the field. A structured literature search was performed across the PubMed (MEDLINE) and Embase databases from inception through April 2026. The search strategy combined Medical Subject Headings (MeSH/Emtree terms) and free-text keywords targeting the themes of colorectal/rectal malignancy; stromal or cellular density characteristics; neoadjuvant treatment modalities; and therapeutic response or clinical outcomes. For the primary clinical evidence synthesised in Table 1, articles were screened against predefined criteria:

- Inclusion Criteria: (1) original peer-reviewed research papers; (2) evaluation of human rectal or colorectal adenocarcinoma; (3) quantification of pre-treatment tumour–stroma ratio (TSR), tumour cell density (TCD), or similar equivalent measurement, specifically in pre-treatment diagnostic biopsies; and (4) correlation of TSR or related features with subsequent pathological response or clinical outcomes such as survival following neoadjuvant therapy.
- Exclusion Criteria: (1) studies evaluating TSR/TCD exclusively in post-treatment surgical resection specimens; (2) review articles, case reports, abstracts, or non-English language publications; (3) in vitro or animal model studies.

### 2.2. Terminology

A barrier to synthesising the current evidence on this topic is the inconsistency in the terminology and measurement thresholds used across studies. While the phenomenon—the balance between neoplastic epithelial cells and the surrounding desmoplastic stroma in the TME—is consistent, the methods of assessment used can vary.

### 2.3. Tumour–Stroma Ratio (TSR) and Tumour Cell Density (TCD)

Traditionally, TSR, as defined by van Pelt et al. [16], represents the percentage of stroma seen within a tumour area, scored in increments of 10% and split into stroma-high (>50%) and stroma-low (</=50%) categories. In most of the literature, TSR is used in this way to refer to the percentage stroma (Table 1). However, some studies have used TSR more literally to mean the ratio of tumour to stroma within the tumour area [17]. So, confusingly, “TSR-high” might refer to a stroma-high tumour or a tumour with a high tumour-to-stroma ratio, which would be stroma-low.

**Table 1:** Summary of clinical evidence assessing pre-treatment tumour–stroma ratio (TSR), tumour cell density (TCD), and epithelium tumour area percentage (ETP) as predictive biomarkers for neoadjuvant therapy efficacy in colorectal cancer.

Study, Year	Relevant Study Aim	Cohort Size (*n*) and Study Design	Cancer Type and Stage	Neoadjuvant Therapy Received	TSR/TCD Scoring Method on Tumour Biopsy H&E Slides	TSR/TCD Cut-Off Value	Endpoint(s)	Key Findings
Shen et al., 2025 (preprint) [18]	To assess if response to nCRT treatment intensification differs by biopsy TCD status	*n* = 414 Retrospective from phase 3 ARISTOTLE multicentre clinical trial	Rectal cancer. Locally advanced (MRI-defined).	Standard nCRT (45 Gy plus capecitabine) or intensified nCRT (45 Gy plus capecitabine plus irinotecan).	AI-automated quantification of TCD. TCD defined as the proportion of tumour cells relative to all classifiable cells in the neoplastic epithelial and tumour-associated stromal region detected by the AI model.	<50% TCD = stroma-high	Disease-free survival, overall survival, and pCR	Stroma-low (high-TCD) patients had significantly improved disease-free survival (HR 0.57, *p* = 0.014), overall survival (HR 0.5, *p* = 0.008), and pCR rates (OR 2.46, *p* = 0.042) with the addition of irinotecan. Stroma-high (low TCD) patients derived no significant survival or pCR benefit from this treatment intensification.
Polack et al., 2025 [19]	To validate the predictive value of biopsy-scored TSR for neoadjuvant therapy response	*n* = 373 Retrospective from two multicentre clinical trials and one local cohort (PROCTOR-SCRIPT trial, *n* = 122; RAPIDO trial, *n* = 154; local LUMC cohort, *n* = 97)	Rectal cancer. Stages I–III.	RAPIDO trial: SCRT (5 × 5 Gy) followed by 6 cycles of CAPOX or 9 cycles of FOLFOX4, compared with standard nCRT PROCTOR-SCRIPT trial: SCRT (5 × 5 Gy) or standard nCRT followed by observation or adjuvant chemotherapy after surgery. Local LUMC cohort: consecutive patients with various (neo)adjuvant therapy regimens.	Manual visual estimation in 10% increments (van Pelt method)	>50% stroma = stroma-high	Pathological response (Mandard TRG; major response = TRG 1–2)	Stroma-high associated with lower major response rates (HR 0.63, *p* = 0.044). Stroma-high was more commonly observed in locally advanced disease compared to early-stage disease.
Callaghan et al., 2025 [20]	To assess the prognostic and predictive value of TCD and TIL density for response to neoadjuvant SCRT	*n* = 253 Retrospective from phase 3 MRC CR07 multicentre clinical trial	Rectal cancer. Resectable.	SCRT (5 × 5 Gy) or straight to surgery	Manual point counting. TCD defined as percentage of points falling on viable tumour cells across the entire tumour area.	<44.2% TCD = stroma-high (cut-off optimised from survival data for SCRT arm)	Overall survival and cancer-specific survival	In the SCRT arm, stroma-high (low-TCD) biopsies were associated with a reduced risk of cancer-related death (HR 0.34, *p* = 0.028). In the control arm, stroma-high (low-TCD) biopsies showed a trend towards a higher risk of cancer-related death (HR 3.29, *p* = 0.109).
Liang et al., 2021 [21]	To investigate the value of biopsy TSR in predicting nCRT response in patients with locally advanced rectal cancer	*n* = 248 Retrospective single-centre	Rectal cancer. Locally advanced, stages I–III.	Standard nCRT: long course, various regimens	Semi-automated, digital quantification of TSR. Percentage stroma area calculated from annotated tumour regions.	<56.3% = stroma-low; 56.3 to 72.8% = stroma-intermediate; >72.8% = stroma-high	Pathological response (AJCC 8th edition TRG; major response = TRG 0–1)	Stroma-high associated with fewer major responders compared to the stroma-low group (48.1% vs. 69.9%, *p* = 0.018). By multivariate analysis, stroma-high was independently associated with a greater chance of no response to nCRT (OR 0.40, *p* = 0.002).
Strous et al., 2022 [22]	To assess whether pre-treatment biopsy TSR is associated with pathological response to nCRT	*n* = 187 Retrospective 2-centre	Rectal cancer. Non-metastatic, stages I–III (81.3% stage III).	Standard nCRT: Long-course radiotherapy (50–50.4 Gy) plus concurrent capecitabine	Manual visual estimation in 10% increments (van Pelt method)	>50% stroma = stroma-high	Pathological response (Mandard TRG; major response = TRG 1–2)	Stroma-high associated with lower probability of achieving major response (OR 0.37, *p* = 0.004); 73.8% of all major responders exhibited a stroma-low phenotype.
Tian et al., 2023 [17]	To explore the predictive value of vimentin expression and TSR for nCRT response	*n* = 159 Retrospective 2-centre	Rectal cancer. Locally advanced, stages II–III.	Standard nCRT: Long-course radiotherapy (45–50.4 Gy) plus capecitabine or 5-FU-based chemotherapy (various regimens)	Manual visual estimation in 10% increments, combined with manual visual vimentin immunohistochemistry scoring (>10% positive tumour cells threshold)	>50% stroma = stroma-high (the authors term this “TSR-low”)	Pathological complete response	Stroma-low (reported as “TSR-high”) associated with a higher likelihood of pCR by univariate analysis (OR 4.97, *p* = 0.002). The combination of vimentin-low/stroma-low was the strongest independent predictor of pCR in multivariate analysis (OR 9.32, *p* = 0.006).
Li et al., 2024 [23]	To determine if biopsy TME characteristics (TSR, mitoses, inflammation, desmoplastic reaction, necrosis, tumour budding) predict neoadjuvant therapy efficacy	*n* = 106 Retrospective 3-centre	Colorectal cancer. Mixed cohort (71 rectal, 35 colon). Stages I–IV.	nCRT or neoadjuvant chemotherapy alone (regimens and number of patients per treatment group not reported)	Manual visual estimation throughout entire biopsy	>50% stroma = stroma-high	Pathological response (AJCC, 8th edition TRG; good response = TRG 0–1)	TSR not significantly associated with response to neoadjuvant therapy (*p* = 0.672). The other H&E-derived features assessed also failed to predict response.
Yim et al., 2022 [24]	To investigate if TME factors (TSR, intratumoural budding, desmoplastic reaction, Klintrup–Mäkinen grade) in pre-treatment biopsies are associated with prognosis and/or neoadjuvant therapy response	*n* = 85 Retrospective single-centre	Colorectal cancer. Mixed cohort (74 rectal, 11 colon *). Stages I–IV.	Various regimens: Standard nCRT and surgery (66 patients) or palliative treatment (19 patients)	Manual visual estimation at a selected tumour hotspot	>50% stroma = stroma-high	Overall survival, cancer-specific survival, disease-free survival, and tumour regression (Dworak TRG; good response = grades 3–4)	TSR not significantly associated with therapy response or tumour regression. High-grade intratumoural budding was the only evaluated feature found to be associated with tumour regression in those that underwent surgery (*p* = 0.049).
Jepsen et al., 2024 [25]	To examine the association between histopathological features in diagnostic biopsies and pathological response to neoadjuvant therapy	*n* = 50 Retrospective 2-centre	Rectal cancer. Stages II–IV.	Various regimens: Standard nCRT (long or short course, *n* = 42), chemotherapy alone (*n* = 7), or radiotherapy alone (*n* = 1)	AI-automated quantification of epithelium tumour area percentage (ETP). ETP calculated as the area of epithelium divided by the total tumour area (epithelium + stroma) × 100%.	ETP analysed as a continuous variable	Pathological response (Mandard TRG; good response = TRG 1–2)	ETP not significantly correlated with response (good response, *p* = 0.937; pCR, *p* = 0.712).

Abbreviations: nCRT = neoadjuvant chemoradiotherapy; pCR = pathological complete response; SCRT = short-course radiotherapy; TCD = tumour cell density; TME = tumour microenvironment; TRG = tumour regression grade; TSR = tumour–stroma ratio. * Different figures are provided in the Abstract (74 rectal/11 colon) and Methods (80 rectal/5 colon) sections of this paper.

The tumour cell density (TCD) typically describes the proportion of neoplastic epithelial cells relative to the total number of cells in a tumour [26]. This can be thought of as the inverse of the conventional TSR measurement (percentage stroma). Other similar measurements, such as the epithelium tumour area percentage (ETP), have also been described [25]. A distinction should be made between assessments of the tumour cell area (spatial fraction) and the tumour cell number, as variations in cell size could affect such measurements. Because neoplastic cells often possess more abundant cytoplasm than surrounding non-neoplastic cells (such as small lymphocytes), visual estimation of the tumour area may overestimate true cellularity. This is a recognised issue in assessing tumour cellular fractions for genomic studies [27]. Whether TSR (an area-based metric) and TCD (a cell count-based metric) are truly interchangeable for predictive purposes remains an unresolved question in the literature. Until comparative studies evaluate whether these metrics yield meaningfully different predictive results in neoadjuvant cohorts, caution should be taken in treating these distinct methodologies as entirely synonymous, even if both broadly categorise tumours into stroma-high and stroma-low phenotypes.

### 2.4. An Evolving Definition for the Digital Pathology Era

Both TSR and TCD quantify the balance between neoplastic cells and the surrounding stroma. However, these metrics are calculated differently, so it remains important to be aware of which definition is being used in studies. As the field moves toward automated quantification, and away from manual stroma-centric evaluation, TCD perhaps represents a more direct output of artificial intelligence (AI) algorithms, which often detect and count individual cells. Furthermore, TCD could offer a more unbiased means of assessing the neoadjuvant therapy response compared to pathologists’ tumour regression grading [28]. It may also be more intuitive to measure a reduction in tumour cell density rather than a relative gain in stroma. Quantifying TCD as a continuous variable preserves data; however, to establish clinical utility, most current evidence relies on defined cut-offs—most commonly, the >50% stroma threshold for stroma-high. Although binary stratification might align with clinical decision-making, researchers must consider potentially non-linear relationships between TSR, neoadjuvant therapy, and clinical outcomes, as has been demonstrated in oesophageal cancer [29]. The >50% threshold appears to be frequently adopted as the default standard due to its widespread use in the existing literature, much of which has looked at TSR in resections for prognosis [8] and not TSR in diagnostic biopsies for predicting neoadjuvant therapy response. Despite its practicality for manual scoring, establishing whether the traditional >50% mark or a new threshold is most appropriate for automated scoring or predicting therapy response remains an important yet unresolved issue.

**Figure 1:**
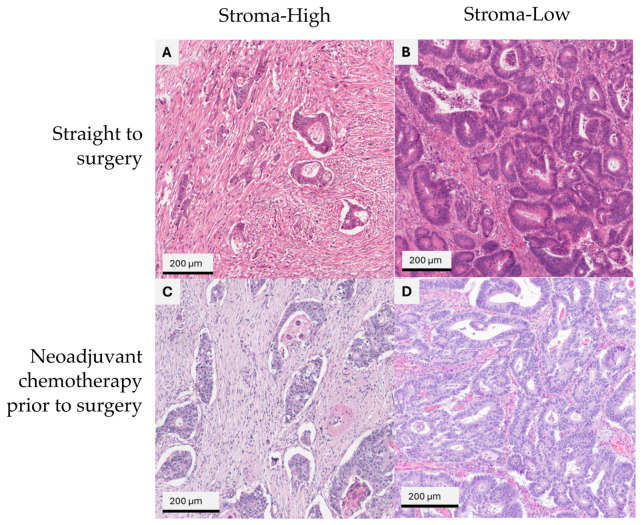
Representative H&E-stained sections from colorectal cancer surgical resections showing variation in stromal content. (**A**,**B**) show tumours from patients who went straight to surgery without neoadjuvant treatment, demonstrating stroma-high (**A**) and stroma-low (**B**) phenotypes. (**C**,**D**) show tumours from patients who received neoadjuvant chemotherapy prior to surgery, showing stroma-high (**C**) and stroma-low (**D**) phenotypes. Stroma-high after neoadjuvant chemotherapy might indicate an initially stroma-high tumour, a significant response to therapy (i.e., regression), or a combination of the two.

Considering TSR and TCD, the use of a ratio and “inverse” measurements presents potential sources of confusion. Beyond semantics, a lack of consistent terminology and measurement could represent a barrier to clinical translation. Clear terminology, such as “stroma-high” (synonymous with conventional TSR-high and TCD-low) and “stroma-low” (conventional TSR-low and TCD-high), could improve consistency in the literature, and are used in this review. Examples of stroma-high and stroma-low colorectal cancers are shown in Figure 1. While an internationally agreed definition is not yet established, studies should be clear in their definitions for TSR. Studies comparing different methods of assessing TSR, and prospective and validated studies, may help to determine which methods yield the greatest prognostic or predictive value, as well as determining what is reproducible and usable in practice.

Van Pelt and colleagues have offered standardised recommendations for manual TSR scoring. They suggest that routine H&E-based methods are rapid, cost-effective, and yield highly reproducible results (kappa 0.68–0.97) [16]. They detail which tissue areas to include and exclude in the assessment (e.g., exclude smooth muscle). They suggest that this method should be applied to the slide with the deepest tumour invasion, although this area is often not visualised in biopsies. Despite these recommendations, varying methodologies and definitions of TSR continue to challenge comparisons between studies [30]. A recent review highlights issues such as variations in cut-off points used for stroma-high tumours (ranging from 40% to 65.5%) and how TSR can be calculated by manual, semi-, or fully automated methods [30]. The relevance of these issues to pre-treatment colorectal cancer biopsies specifically remains relatively unexplored.

### 2.5. Manual Scoring

To date, most of the literature relies on manual TSR scoring methods, such as visual estimation or point counting. These assessments may evaluate the whole tumour area or selected tumour regions to estimate the stroma percentage.

The visual estimation of TSR, such as the Mesker approach [31], is a relatively straightforward and accessible technique with minimal cost implications. However, such methods can be limited by their semi-quantitative nature and interobserver variability [30]. To mitigate these issues and improve the reproducibility of manual visual estimation, educational initiatives could prove useful. For example, in the UNITED study, which involved a cohort of colon cancer patients that had not received any neoadjuvant therapy, a quality-controlled e-learning program to train pathologists in the Mesker scoring method helped to achieve good interobserver agreement (kappa of >0.70) [8]. Quantitative manual approaches like point counting can yield improved interobserver agreement but are likely to be highly time-consuming and may still only assess a sample of the tumour area. Although point counting may be impractical for routine diagnostics, it provides a reproducible quantitative assessment for research contexts. There is a lack of standardisation in how manual TSR scoring is achieved, with variations in protocols across studies, most prominently including differences in selecting regions of interest [30]. There is no consensus on the optimal tumour region and region size for TSR scoring, with practices varying across the whole tumour, the infiltrative edge, or regions of the highest stroma. This variability introduces selection bias and complicates cross-study comparisons. Different regions of interest can also generate different cut-off values when trying to dichotomise cohorts into good and poor outcomes [30]. Recent advancements in digital pathology are beginning to address some of these problems. For instance, in the UNITED cohort, an automated AI-based TSR quantification algorithm has been validated, and established that a 1 mm region of interest could maximise prognostic accuracy. Interestingly, this method yielded an optimal automated cut-off (77%) that differed from the conventional 50% threshold [32].

### 2.6. Automated Scoring

**Figure 2:**
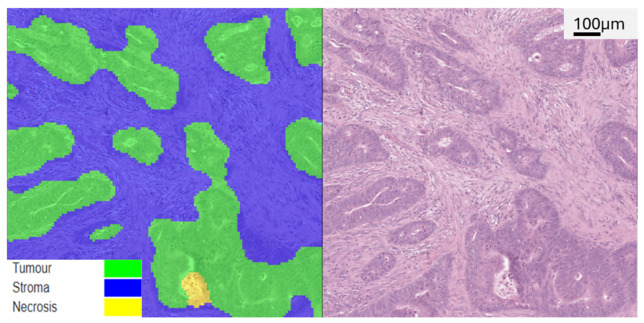
(**Left**): A region of colorectal adenocarcinoma in a surgical resection specimen with an overlay delineating areas of tumour (green), stroma (blue), and necrosis (yellow). This overlay was produced using a deep learning artificial intelligence model, illustrating the potential for automating TSR measurements. (**Right**): The corresponding H&E-stained colorectal cancer section showing epithelial adenocarcinoma cells set in a desmoplastic stroma.

Semi- or fully automated approaches to TSR/TCD quantification, including AI deep learning tools (Figure 2), may provide better standardised scoring on a large scale [30], as well as offering potential to be combined with other AI-derived biomarkers on H&E slides, such as the tumour-infiltrating lymphocyte density [33]. Liang et al. used a semi-automated method of quantification to show that TSR in locally advanced rectal cancer biopsies was a reproducible independent predictor of response following nCRT, with three defined cut-off categories [21]. Similarly, while not directly assessing TSR, Lafarge et al. demonstrated that deep learning can be used to classify rectal cancer biopsies by consensus molecular subtype and linked high stromal content in one subtype with poor outcomes following nCRT [34]. Deep learning models have demonstrated over 95% accuracy in determining TSR, yet human-to-AI agreement remains low (kappa 0.239 to 0.472) [30]. This suggests that, while deep learning-based models excel in patch- and pixel-based classification, concordance could be limited by the manual selection and evaluation of slides and regions of interest. Without standardised protocols, the selection bias and variability inherent in the human “gold standard” limit the generalisability of these algorithms, underscoring the need for extensive clinical validation before automated TSR assessment can be widely adopted.

Slide scanning for digital TSR analysis can be more time-consuming than manual scoring using a microscope. However, this logistical barrier is diminishing, with many centres transitioning to fully digital pathology workflows. If validated, AI tools for the automatic quantification of TSR could therefore be quickly and reliably implemented clinically. However, this would still come with challenges, such as those around system integration, quality control, and regulatory approval.

## 3. The Clinical Evidence for TSR as a Predictive Biomarker in Rectal Cancer

The use of pre-treatment TSR to predict neoadjuvant therapy response is an emerging area of research. Evidence suggests that TSR in diagnostic biopsies may serve as a surrogate marker for therapeutic sensitivity or resistance, although findings vary across study cohorts and treatment modalities (Table 1).

### 3.1. Evidence Linking High Stroma to Poor Pathological Response

Several retrospective studies suggest that stroma-high rectal cancers may exhibit resistance to standard nCRT (Table 1). In a recent large, multicentre study validating TSR in pre-treatment biopsies, Polack et al. demonstrated that stroma-high tumours (>50% stroma) had significantly lower major response rates to neoadjuvant therapy compared with stroma-low tumours (HR 0.63, 95% CI 0.41–0.99; *p* = 0.044) [19]. This study utilised a local cohort alongside cohorts from the PROCTOR-SCRIPT and RAPIDO trials, providing supporting evidence for the hypothesis that the stromal compartment can mediate treatment resistance.

These findings are supported by Strous et al. [22], who report that stroma-high in pre-treatment biopsies was associated with a significantly lower probability of achieving a major pathological response (OR 0.37, 95% CI 0.19–0.73, *p* = 0.004). In their two-centre cohort, 73.8% of major responders were classified as stroma-low, suggesting that this phenotype supports tumour regression [22]. Similarly, using a semi-automated digital quantification method, Liang et al. observed significantly more major responders among stroma-low tumours (69.9%) compared with the stroma-high group (48.1%, *p* = 0.018) [21]. In their multivariate analysis, TSR was the only independent pre-treatment predictor of response to nCRT [21].

The potential predictive utility of TSR could be enhanced when combined with other stromal markers, such as vimentin, which has previously been demonstrated as a prognostic biomarker [35]. Tian et al.’s work supports the predictive value of TSR but also highlights the added value of vimentin [17]. They observed that stroma-low (reported in the study as “TSR-high”) was associated with a significantly higher likelihood of achieving pCR with nCRT (OR 4.97, 95% CI 1.93–15.43, *p* = 0.002). Conversely, vimentin-high tumours were associated with lower odds of pCR when compared with vimentin-low tumours (OR 0.26, 95% CI 0.10–0.60, *p* = 0.002). In a multivariate analysis, the combination of low vimentin expression and a stroma-low phenotype was the strongest independent predictor of pCR (OR 9.32, 95% CI 2.29–63.6, *p* = 0.006) [17]. However, while this approach may be informative, it requires additional staining with vimentin. Another combined biomarker that can be assessed on routine H&E slides with potential for predicting response to therapy in colorectal cancer is the Glasgow Tumour Microenvironment Score (GMS) [36]. The GMS, which incorporates the tumour stroma percentage and peritumoural inflammation (using the Klintrup–Mäkinen grade), may help to predict response to specific adjuvant chemotherapy regimens in colorectal cancer; patients with a favourable microenvironment (GMS 0) derived a disease-free survival benefit from FOLFOX over CAPOX, whereas those with a stroma-high or immune-poor phenotype did not [36]. Despite evaluating resection slides in the adjuvant chemotherapy setting, rather than pre-treatment diagnostic biopsies, their findings support the role of the stroma in modulating chemosensitivity and lend weight to its potential utility as a predictive biomarker. 

While all the studies outlined in Table 1 were conducted retrospectively, some [18,19,20] may arguably represent more robust evidence as they are post hoc analyses of large, prospective randomised controlled trial datasets (ARISTOTLE, RAPIDO/PROCTOR-SCRIPT, and MRC CR07 respectively). In contrast, the remaining retrospective cohort studies may be more susceptible to selection bias, heterogeneity, and confounding factors.

### 3.2. Conflicting Evidence and Methodological Heterogeneity

Despite the positive findings in larger cohorts, several smaller studies have failed to establish a predictive link between biopsy TSR and treatment response. A retrospective multicentre study by Li et al. investigated multiple TME features, including TSR, tumour budding, and desmoplastic reaction, but found that none, including TSR (*p* = 0.672), were significantly associated with a pathological response to neoadjuvant therapy [23]. Similarly, Yim et al. reported that, while intratumoural budding was predictive of recurrence and regression, TSR in pre-treatment biopsies was not associated with neoadjuvant therapy response [24]. These discrepancies could be attributed to the specific cohorts examined and heterogeneity in study designs. Both Li et al. [23] and Yim et al. [24] evaluated mixed cohorts of patients with colon and rectal cancers, across a wide range of stages, who underwent varied neoadjuvant treatment regimens. To assess TSR, they utilised visual estimation across the biopsy or selected regions of interest. Most studies assess response using a pathological regression grading system (e.g., Mandard or Dworak), although some evaluate survival outcomes. Although tumour regression grading is simple, clinically relevant, and correlates with survival [3,12], well-documented intra- and interobserver variability [37,38] mean that survival outcomes may offer a more reliable measure of response.

Jepsen et al. used a deep learning-based digital approach to assess the epithelium tumour area percentage (ETP) in rectal cancer biopsies [25]. Unlike other studies using or establishing specific cut-offs, they analysed ETP as a continuous variable. While they found that higher densities of CD8+ lymphocytes predicted a favourable pathological response, ETP did not correlate with response to neoadjuvant therapy. Similarly to the cohorts evaluated by Li [23] and Yim et al. [24], this study was limited by a relatively small sample size (*n* = 50) and a heterogenous mix of neoadjuvant treatment regimens. This clinical variation inevitably limits the specificity and generalisability of their conclusions. Shen et al. also employed a deep learning-based approach and suggest that AI measurements may overcome the limitations of manual scoring. They helpfully validated their AI model against manual annotation, achieving strong concordance (concordance correlation coefficient = 0.83), and argue that AI-driven quantification is more robust, scalable, and cost-efficient than manual methods [18].

### 3.3. TSR as a Regimen-Specific Predictive Biomarker

Importantly, several of the studies that showed no association between TSR and outcomes evaluated heterogenous cohorts without stratifying patients by treatment regimen [23,24,25]. In contrast, studies demonstrating significant predictive value for TSR often utilised pure cohorts of patients receiving more consistent neoadjuvant protocols, such as standard nCRT. Because the predictive value of TSR is likely to be dependent on the specific neoadjuvant regimen administered, combining distinct therapeutic modalities into a single unstratified analysis risks potentially diluting regimen-specific predictive signals. While a stroma-high phenotype appears to be generally associated with a poorer response to standard nCRT, evidence suggests a different interaction in the context of short-course radiotherapy (SCRT) [20]. In the MRC CR07 clinical trial, a stroma-high phenotype (low pre-treatment biopsy TCD) showed potential as a predictive biomarker for SCRT benefit. Within the SCRT arm, stroma-high patients had a reduced risk of cancer-related death compared to stroma-low patients (HR 0.34, *p* = 0.028). This contrasts with the poor underlying prognosis typically associated with stroma-high tumours [20]. Owing to a short interval to surgery, no pCRs were observed in this trial. However, the survival findings contrast with those expected in the standard nCRT setting and suggest that stroma-high tumours may respond more favourably to specific radiation protocols. Furthermore, Shen et al. raise the possibility of using TCD as a biomarker for treatment escalation [18]. In a post hoc analysis of the ARISTOTLE trial, the AI-automated quantification of TCD revealed a significant treatment–TCD interaction. Patients with stroma-low (TCD-high) tumours derived a significant survival benefit from the addition of irinotecan to standard nCRT, whereas the stroma-high (TCD-low) group did not benefit from this escalation [18].

While both manual and automated assessments have linked stroma-high phenotypes to a poor pathological response in standard nCRT, it appears that this relationship may not translate to all neoadjuvant regimens. The contrasting findings in SCRT and intensified chemotherapy underscore the necessity of treating TSR as a regimen-specific biomarker rather than a universal predictor of resistance. The relationship between H&E-derived TSR and neoadjuvant immunotherapy responses has not yet been characterised. These are important considerations in terms of using TSR as a biomarker in a personalised medicine approach to help select the most appropriate management strategy for a given patient.

### 3.4. Study Limitations

The outlined studies are retrospective, feature heterogeneous trial or study designs, and utilise variable treatment regimens and endpoints, necessitating cautious interpretation of TSR’s clinical readiness. A significant limitation across the current literature is the lack of robust external validation for many of the proposed metrics. Future research must focus on prospective validation to determine if this simple histological marker can reliably guide complex therapeutic decisions. Additionally, the inconsistent use of pathological response grading systems, alongside variable definitions and cut-offs for TSR/TCD, complicates direct comparisons between cohorts.

Beyond study design, biopsy-based assessment carries inherent limitations. Endoscopic biopsies may incompletely represent the wider TME due to intratumoural spatial heterogeneity. The superficial luminal aspect of the tumour may differ morphologically and biologically from the invasive front, where desmoplastic reactions are often more prominent. There is no consensus on minimum biopsy adequacy, such as the area or number of tumour cells required to provide a reproducible and clinically meaningful TSR assessment. A mucinous tumour morphology and extensive necrosis may also complicate TSR assessment by both manual and automated methods, underscoring the need for clear protocols.

## 4. The Biological Rationale for TSR as a Predictive Biomarker

The potential predictive value of TSR is likely underpinned by a complex interplay between physical, cellular, and molecular mechanisms within the TME. A stroma-high microenvironment incorporates features such as fibroblast activation, a dense extracellular matrix, altered perfusion, and immune suppression [39]. It is challenging to separate the biological mechanisms that underpin TSR’s potential predictive utility from its established prognostic value, as features that may be associated with more aggressive tumour behaviour might interact differently with certain neoadjuvant treatment modalities.

### 4.1. Mechanisms of Stroma-Mediated Treatment Resistance

Stroma-high tumours frequently correlate with the consensus molecular subtype 4 (CMS4) mesenchymal phenotype, which is characterised by prominent stroma and TGF-beta activation of cancer-associated fibroblasts (CAFs) [34,40,41]. Inflammatory and myofibroblastic CAFs actively drive extracellular matrix remodelling and promote epithelial-to-mesenchymal transition [40,41]. Furthermore, specific molecular features such as the enrichment of Galectin-1 in stroma-high tumours can promote tumour cell survival, invasion, and stem-like behaviour, all of which are associated with intrinsic resistance to standard chemoradiotherapy [42,43].

Structurally, extracellular matrix remodelling and increased tissue stiffness may create a physical barrier to treatment. A dense desmoplastic stroma is thought to compress the tumour vasculature and increase interstitial fluid pressure, which could impair the delivery of chemotherapeutic agents and contribute to regions of hypoxia [44]. Given the reliance on tissue oxygen to generate reactive oxygen species and free radicals to induce DNA damage, this may further reduce the efficacy of radiotherapy [44].

A dense tumour stroma may also dictate the immunological landscape, frequently correlating with an immunosuppressive TME. TGF-beta signalling and a dense extracellular matrix could act as a combined chemical and physical barrier, helping to exclude cytotoxic immune cells from the tumour [41,45]. Stroma-high colon cancers have also shown increased infiltration of immunosuppressive regulatory T cells [46]. Radiotherapy-induced changes in stromal and immune gene expression further highlight the dynamic interplay between treatment and the TME [28].

### 4.2. Radiobiology and Regimen-Specific Sensitivities

The described features provide some biological rationale for why stroma-high tumours often respond poorly to standard neoadjuvant therapy. The finding from the CR07 trial that stroma-high tumours might derive a survival benefit from SCRT might appear counterintuitive when compared with the standard nCRT literature. While this discrepancy could represent a statistical artefact in a retrospective analysis utilising an optimised cut-off, it might also be explained by the distinct radiobiology and immunological impacts of the contrasting regimens.

Standard long-course nCRT relies on the delivery of smaller radiation doses per fraction over many weeks, with concurrent radiosensitising chemotherapy. This timeline is thought to exploit the classic “Rs” of radiobiology, allowing for the gradual reoxygenation of hypoxic cells and their redistribution into more radiosensitive stages of the cell cycle. Standard fractionated radiotherapy aims to minimise radiation-induced vascular damage, and relies on intact vasculature to facilitate reoxygenation, DNA damage, and drug delivery [47].

In contrast, SCRT delivers a higher dose of radiotherapy per fraction over a shorter interval. While conventional smaller-dose fractions preserve vascular function, larger fractional doses can trigger acute and severe vascular damage, by inducing endothelial cell apoptosis in the tumour microvasculature [47]. Secondary ischaemic cell death may help to circumvent the intrinsic cellular resistance and chronic hypoxia that might otherwise protect stroma-high tumours from standard fractionated nCRT [47]. A recent longitudinal clinical study has also shown how these distinct regimens translate into different immunological landscapes. Serial sampling of patients undergoing neoadjuvant treatment showed that standard long-course radiotherapy induces the prolonged depletion of circulating lymphocytes. Meanwhile, SCRT is significantly less systemically lymphodepleting and induces more frequent increases in intratumoural CD8 and FOXP3+ T cell infiltration at 2 and 6 weeks post-treatment [48]. By preserving systemic lymphocytes and boosting local cytotoxic T cell activity, SCRT may help to better elicit a systemic anti-tumour immune response and reprogram the immunosuppressive stroma.

Much like varying radiotherapy fractionation, the use of specific systemic agents highlights how the predictive value of TSR is likely to be regimen-dependent. For example, the transcriptional profiling of rectal cancers by Mahmood et al. showed that, while high stromal signatures predicted resistance to standard fluoropyrimidine-based chemoradiotherapy, this resistance could be attenuated by the addition of oxaliplatin [49]. Conversely, recent findings indicate that treatment intensification with irinotecan provides a differential survival benefit specifically for stroma-low tumours [18]. These data suggest that the biological properties of the stroma may dictate sensitivity to specific agents, highlighting the potential of TSR to guide personalised treatment strategies.

## 5. Future Directions

The evidence reviewed suggests that, beyond its established prognostic value, TSR is a promising candidate biomarker for predicting response to neoadjuvant therapy in rectal cancer. Translating this potential into clinical practice requires better standardised assessment methods and robust prospective validation in large cohorts. Digital pathology and AI will be central to this effort; deep learning frameworks have already demonstrated the feasibility of automatically and reproducibly quantifying features like immune infiltrates from routine H&E slides to predict survival in this setting [50]. Applying similar automated workflows to TSR could streamline its assessment. A future translational workflow might provide an AI-quantified TSR, which could then be integrated with spatial profiling or deep learning-derived features, such as the immune context or molecular subtypes. Combining these data with other clinicopathological or imaging metrics could yield multimodal predictive models to help stratify patients and inform treatment selection. While such workflows might be informative, they must ultimately serve as tools to facilitate shared decision-making, ensuring that patient choice, priorities, and quality of life considerations remain at the forefront of personalised care.

### 5.1. Standardisation and Digital Pathology

The main hurdles in the clinical implementation of routine TSR/TCD assessment include methodological inconsistency and a lack of prospective validation. Specifically, variations in scoring methods, regions of interest, and cut-off thresholds limit reproducibility and cross-study comparisons. Clear terminology and a consensus on the most appropriate methods for the routine evaluation of TSR/TCD are essential. Shen et al. [18] demonstrate the feasibility of generating TCD data using an AI framework and its clinical relevance in rectal cancer. Moving towards automated, objective quantification will help to address problems with interobserver variability and reproducibility. However, the widespread clinical implementation of these automated tools may be hindered by broader digital pathology challenges, including scanner and staining variability, algorithmic explainability, validation, monitoring, integration into existing workflows, regulation, and quality assurance.

### 5.2. Immune Biomarkers and the dMMR/MSI-H Context

While the stromal content might act as a physical and biological barrier to therapy, the immune context is also likely to be important. A high pre-treatment CD8+ TIL density appears to represent a predictive marker for pCR and better survival in rectal cancer treated with neoadjuvant therapy [51,52,53]. Because stroma-high tumours frequently exhibit an immune-exclusive phenotype [41], combining TSR assessment with immune cell infiltration data could refine predictive accuracy. For example, Ravensbergen et al. showed that the combined assessment of TSR and immune cell infiltrates could predict response to immune checkpoint inhibitors (ICIs) in colon cancer [46], supporting the concept of the stromal architecture influencing immune cell trafficking and therapeutic efficacy [54].

The recent shift towards neoadjuvant immune checkpoint inhibitors in mismatch repair-deficient (dMMR) and microsatellite instability-high (MSI-H) rectal cancer, while representing a minority of early-stage rectal tumours, highlights the need to understand how the broader TME influences immunotherapy efficacy. A recent landmark study demonstrated a 100% clinical complete response rate in locally advanced dMMR rectal cancers treated with six months of neoadjuvant dostarlimab, allowing these patients to safely undergo non-operative management [55]. This response rate suggests that a predictive biomarker such as TSR might be redundant in this particular setting, but, because this therapeutic paradigm is so new, translational studies investigating stromal features within these cohorts remain sparse. The relationship between H&E-derived TSR and neoadjuvant immunotherapy responses in rectal cancer has not yet been characterised and therefore represents a research gap in this field.

### 5.3. Multimodal and Non-Invasive Biomarkers

While TSR promises to be a simple and useful biomarker, multimodal biomarkers may offer further predictive value—for example, incorporating other features such as tumour budding or PD-1+ cell density [52,53,56]. Beyond morphological and immune features, the application of deep learning to routine H&E slides is rapidly expanding to predict molecular signatures. For instance, a deep learning framework combining conventional imaging features with nuclear segmentation data has been shown to accurately predict microsatellite instability and tumour mutational burden in colorectal and gastric cancers [57]. Integrating automated TSR quantification with deep learning-derived features may eventually facilitate the development of multimodal, predictive models that capture both the structural and genomic landscape of a tumour.

With some studies investigating non-invasive surrogates for stromal content, a multimodal biomarker for predicting therapy response in rectal cancer might not rely on H&E slides alone. For example, pre-treatment MRI could be used to predict pathological TSR scores [58], potentially capturing the heterogeneity of the whole tumour volume and mitigating the risks of sampling bias inherent in endoscopic biopsies. Others are exploring stratifying patients by response, using the magnetic resonance tumour regression grade, to escalate or de-escalate therapy [59].

## 6. Conclusions

The investigation of TSR in rectal cancer is currently shifting from its established role as a prognostic indicator in surgical resections to a potentially predictive biomarker in pre-treatment biopsies. As evidence emerges that appears to support its ability to predict neoadjuvant therapy response, TSR could offer a simple, cost-effective, and biologically plausible tool. There appears to be biological rationale for stroma-mediated therapy resistance, but discrepancies in clinical data are likely rooted in methodological heterogeneity and a failure to account for regimen-specific interactions. Future clinical studies should focus on prospective, regimen-specific validation. Furthermore, the integration of digital pathology and AI-assisted quantification will be key to improving reproducibility and standardisation in measuring TSR. Ultimately, automated TSR quantification could offer a practical tool to refine clinical decision-making—for instance, helping to stratify patients for organ-preserving strategies or treatment intensification.

## Data Availability

No new data were created or analysed in this study.
